# The Impact of Self-Sacrificial Leadership on Employee Creativity: A Moderated Mediation Model in the Post-Pandemic Chinese Service Sector

**DOI:** 10.3390/bs15030373

**Published:** 2025-03-16

**Authors:** Yong Liu, Woo-Sung Choi, Wenxian Wang, Seung-Wan Kang

**Affiliations:** 1 Department of Business Administration, College of Business, Gachon University, Seongnam-si 13120, Republic of Korea; liuyong@gachon.ac.kr (Y.L.); wschoi2@gachon.ac.kr (W.-S.C.); 2 School of Innovation and Entrepreneurship, Wannan Medical College, Wuhu 241002, China

**Keywords:** self-sacrificial leadership, autonomy, competence, creative behavior, self-determination theory

## Abstract

Since the end of the COVID-19 pandemic, the economies and trade of many countries have recovered. Executives in various countries have demonstrated self-sacrificial leadership in response to the pandemic by reducing their salaries, emphasizing solidarity and social responsibility, and setting a good example of how companies can weather a storm. In this context, this study investigated the effects of self-sacrificial leadership on China’s service industry. Based on self-determination theory, a moderated mediation model was constructed to investigate the impact on the service industry in China. Surveys were conducted with 472 employees from three service companies located in Hebei Province, China, to construct a research model of self-sacrificial leadership, autonomy, competence, and creative behavior. We employed a two-wave approach with a one-month interval between waves for data collection. Statistical analysis and hypothesis testing were performed using STATA 18.0. Intriguingly, as competence intensifies, the mediating role of autonomy between self-sacrificial leadership and creative behavior intensifies. Our study underscores that increasing competence is imperative for organizations to harness self-sacrificial leadership and boost creative behavior via autonomy.

## 1. Introduction

During the COVID-19 pandemic, many business leaders prioritized the interests of others in a display of self-sacrifice (F. Yang et al., 2023). In response to the pandemic impact, executives in many countries also cut their salaries to establish a solid foundation for their companies to successfully overcome the challenge. This brought the academic concept of self-sacrificial leadership to public attention.

Therefore, despite the increasing body of research on self-sacrificial leadership in recent years, several critical gaps remain in the literature. First, while existing studies have explored the outcomes of self-sacrificial leadership, its effects in crisis situations remain largely unexplored (H. Zhang & Ye, 2016). Second, autonomy has been recognized as a key psychological mechanism in leadership research; however, its mediating role in the relationship between self-sacrificial leadership and creative behavior has yet to be thoroughly investigated. Third, the role of employee competence remains insufficiently understood. Bridging these gaps is essential for advancing both theoretical and practical understanding of how self-sacrificial leadership fosters innovative behavior in times of crisis.

Self-sacrificial leadership involves leaders partially or wholly forgoing or postponing the distribution of rewards, personal benefits, privileges, or exercising power (permanently or temporarily) for the group’s benefit (Van Knippenberg & Van Knippenberg, 2005). This type of leadership behavior boosts followers’ morale and motivation by positively influencing their emotions. When leaders demonstrate selfless behavior instead of selfishness, it fosters a more positive outlook among members and increases their willingness to collaborate with the leader (De Cremer, 2006). Employees’ creative behavior is essential for sustaining the dynamism and fundamental competitiveness of an organization and presents a significant test for organizations (Xu et al., 2022; Wang et al., 2022). Amabile defines creative behavior as the process of generating novel and suitable ideas, processes, products, or solutions (Amabile, 1983). It is widely viewed as a critical precursor to innovation and the successful implementation of creative ideas. Moreover, creative behavior is increasingly acknowledged as a crucial element of effectiveness and a driving force for innovation, expansion, and social advancement across a wide range of occupations and institutions (George, 2007; Wang et al., 2024a). Amabile et al. discovered that positive emotional responses characterized by pleasure are positively associated with creative behavior (Amabile et al., 2005). Furthermore, Hoogervorst et al. found that leaders who prioritize the common good over personal gain are better able to motivate and inspire employees, ultimately impacting team or organizational performance (Hoogervorst et al., 2012).

Leaders play a pivotal role in shaping employees’ work environments and facilitating conditions that support their fundamental psychological needs (Deci et al., 1989). Among various leadership styles, self-sacrificial leadership has garnered attention for its ability to enhance employees’ intrinsic motivation by fostering a heightened sense of purpose and responsibility. Drawing on self-determination theory (SDT), this study explores the mechanisms through which self-sacrificial leadership fosters employees’ creative behavior by satisfying their psychological needs for autonomy and competence. Competence pertains to an individual’s belief in their ability to effectively harness motivation, cognitive resources, and strategic actions to navigate and meet situational demands.

Autonomy serves as a critical antecedent of creativity, as it enables employees to engage in independent, self-directed exploration of novel ideas (X. Zhang & Bartol, 2010). Meanwhile, competence reinforces employees’ confidence in pursuing creative endeavors and experimenting with innovative approaches (Bandura, 1977). Building upon this theoretical foundation, this study examines the interactive effects of autonomy and competence in shaping the relationship between self-sacrificial leadership and creative behavior within the SDT framework.

In today’s rapidly evolving business environment, employees’ creative behavior is essential for organizational survival and growth. While self-sacrificial leadership has been recognized as an influential leadership style that can enhance employees’ creative behavior, research in this area remains limited and fragmented (H. Zhang & Ye, 2016). Although some studies have examined the relationship between self-sacrificial leadership and creative behavior (Liang & Fan, 2020), no prior research has explained the underlying mechanisms of this relationship through the lens of self-determination theory (SDT). More importantly, to the best of our knowledge, no empirical study has examined these mechanisms in the service industry. Given the unique characteristics of the service industry, such as high emotional labor demands and intensive interpersonal interactions, further empirical investigation is warranted to understand the role of self-sacrificial leadership in shaping employees’ creative behavior.

To bridge this research gap, this study focuses on employees in the service industry and extends self-determination theory by analyzing the relationship between self-sacrificial leadership and employees’ creative behavior. Specifically, we investigate the mediating role of autonomy and the moderating role of competence, providing a more structured theoretical framework to explain how self-sacrificial leadership fosters creativity. By clarifying these mechanisms, this study advances theoretical discourse on leadership and creativity while offering practical insights into leadership development and innovation management in service-oriented organizations.

## 2. Theoretical Background and Hypotheses

### 2.1. Self-Determination Theory

According to SDT, human motivation is driven by various factors that can be categorized as intrinsic or extrinsic. Intrinsic motivation is not about solving external demands or physiological needs but is a kind of motivation driven by the nervous system. Its purpose is to encourage individuals to explore and interact with the environment and bring a sense of efficacy through their behaviors, rather than simply through external rewards or survival needs (White, 1959). Extrinsic motivation refers to a type of motivation that is driven by external rewards or punishments. With regard to extrinsic motivation, people identify with the value of an activity and ideally integrate it into their sense of self (Deci & Ryan, 2008). The three fundamental demands under SDT are competence, autonomy, and relatedness (Deci et al., 2017; Deci & Ryan, 2000, 2008).

Competence pertains to an individual’s belief in their ability to effectively harness motivation, cognitive resources, and strategic actions to navigate and meet situational demands. Autonomy encompasses the capacity to define and pursue personally meaningful goals, grounded in a deep understanding of and alignment with one’s values and sense of self. Relatedness reflects the fundamental human need to establish meaningful social connections, characterized by feelings of mutual care, affection, and belongingness (Deci et al., 1989).

Therefore, organizations should promote employee competence, autonomy, and relatedness to enhance employee motivation. Using SDT, our research meticulously examined the mechanism by which members’ self-sacrificial leadership, autonomy, and competence influence their creative behavior.

### 2.2. Relationship Between Self-Sacrificial Leadership and Creative Behavior

Self-sacrificial leadership is widely regarded as a fundamental characteristic of various effective leadership styles, fostering team members’ attachment and commitment to their leader. When leaders engage in self-sacrificial behaviors, team members tend to perceive them as highly effective in achieving collective goals, which, in turn, enhances their emotional attachment to the leader (Chen et al., 2020). This heightened attachment and commitment not only strengthens employees’ engagement with their work but also serves as a catalyst for creative behavior.

At its core, self-sacrificial leadership entails voluntarily assuming additional responsibilities and forgoing personal rewards for the benefit of the team (Chen et al., 2020). This act of social exchange fosters reciprocal motivation among team members, encouraging them to actively engage in creative problem-solving and innovation. Furthermore, self-sacrificial leaders play a pivotal role in aligning employees’ organizational identification with the company’s broader mission and objectives (De Cremer et al., 2004). Given that a strong sense of organizational identification has been shown to enhance employees’ motivation to invest their cognitive and emotional resources into their work, it stands to reason that self-sacrificial leadership can significantly contribute to fostering creative behavior. Extensive research has explored the relationship between leadership styles—such as supportive vs. controlling leadership and transformational leadership—and employees’ creative behavior (X. Zhang & Bartol, 2010). Given that self-sacrificial leadership is a key component of charismatic and transformational leadership (Halverson et al., 2004), a connection between self-sacrificial leadership and employees’ creative behavior is both theoretically and empirically plausible.

Shalley and Gilson (2017) define creative behavior as the process of generating novel and useful ideas and producing innovative, appropriate outcomes that contribute to an organization’s innovative efforts. In the workplace, creative behavior is fundamental to fostering high-performance organizations (Carmeli & Spreitzer, 2009). However, creativity does not occur in isolation; it thrives in a work environment where employees collaborate, share ideas, and operate in an interdependent setting (Opoku et al., 2023). Self-sacrificial leadership fosters precisely such an environment, thereby facilitating creative behavior and supporting organizational growth into a high-performance entity. Existing research has established the positive influence of self-sacrificial leadership on various organizational and employee outcomes, including organizational commitment, cooperative behavior, organizational citizenship behavior (OCB), prosocial actions, subordinate responsibility, and teamwork engagement. For instance, De Cremer et al. demonstrated that self-sacrificial leadership enhances organizational commitment, employees’ cooperative behavior, OCB, and prosocial actions (De Cremer et al., 2004; De Cremer & Van Knippenberg, 2005). Li et al. (2016) further confirmed its positive effect on subordinate responsibility, while Chen et al. (2020) provided empirical evidence for its role in fostering teamwork commitment.

Given that creative behavior is inherently intertwined with these constructs, and based on the well-established relationships between self-sacrificial leadership and such employee outcomes, it is reasonable to posit that self-sacrificial leadership also has a significant positive impact on employees’ creative behavior. Therefore, this study proposes the following hypothesis:

**Hypothesis** **1.** *Self-sacrificial leadership is positively associated with creative behavior.*

### 2.3. Mediating Role of Autonomy

Autonomy refers to an individual’s ability to regulate their experiences and actions independently, as well as their intrinsic motivation to align their activities with their sense of self. It is fundamentally defined as the innate and natural tendency to pursue personal interests and apply one’s abilities (Wang et al., 2024b). Autonomy is a critical component of healthy psychological functioning, and experiencing autonomy is essential for intrinsic motivation and engagement. When individuals operate in environments that foster autonomy, their intrinsic motivation, satisfaction, and overall well-being are significantly enhanced (Deci & Ryan, 2000).

Autonomy is particularly crucial in the context of creative performance, as it enables individuals to engage in voluntary, authentic, and self-directed creative processes (Liang & Fan, 2020). Higher levels of autonomy contribute positively to creative behavior by strengthening intrinsic motivation, encouraging individuals to explore diverse ideas and approaches, and enhancing their capacity to solve problems in more innovative ways (Lee et al., 2021).

According to self-determination theory (SDT), the fulfillment of autonomy is a key determinant of employee motivation and commitment to tasks (Ryan & Deci, 2006). Leaders play a pivotal role in satisfying employees’ psychological needs, and autonomy, in particular, is a fundamental prerequisite for fostering intrinsic motivation (Deci et al., 1989). Self-sacrificial leadership, which serves as a powerful source of inspiration, deeply influences employees’ emotions and motivation, elevating their sense of purpose and optimism (De Cremer, 2006). Moreover, self-sacrificial leaders relinquish personal privileges in favor of their employees, empower them with autonomy, and fulfill their psychological needs, thereby cultivating intrinsic motivation and fostering creative behavior (Hammond et al., 2011).

Given these theoretical underpinnings, this study seeks to demonstrate—through the lens of self-determination theory—that autonomy mediates the relationship between self-sacrificial leadership and creative behavior. Supporting this perspective, De Cremer et al. (2006) conceptualized self-sacrifice as an empowering leadership mechanism. Similarly, Guo et al. (2021) identified a positive association between job autonomy and employee creativity in the hotel industry in Pakistan. Samimi Dehkordi et al. (2024) further reinforced the role of autonomy as a key enabler of creative behavior, emphasizing its importance in fostering innovative workplace dynamics. Therefore, this study proposes the following hypothesis:

**Hypothesis** **2.** *Autonomy positively mediates the relationship between self-sacrificial leadership and creative behavior.*

### 2.4. Moderating and Moderated Mediation of Competence

Competence refers to an individual’s belief in their ability to effectively complete tasks and overcome challenges (Bandura, 1977). This perception plays a crucial role in shaping the types of challenges or activities individuals feel confident in pursuing and successfully accomplishing (Walumbwa et al., 2011). A strong sense of competence is integral to creative productivity and the discovery of new knowledge (Bandura, 1977; Tierney & Farmer, 2002), as highly competent individuals tend to exhibit greater engagement and innovation in the creative process. Moreover, competence serves as an essential personal resource, reflecting an individual’s self-perceived ability to execute specific behaviors effectively. Employees with a strong sense of competence are more likely to foster positive relationships with colleagues (Wang et al., 2021) and demonstrate key professional attributes, including knowledge, skills, attitudes, and values, which enhance their capacity to perform their professional responsibilities successfully (Wang et al., 2024a; Takase et al., 2015).

According to self-determination theory (SDT) (Ryan & Deci, 2000), individuals are intrinsically driven to fulfill their psychological needs, which are closely associated with their creative behavior (Van Den Broeck et al., 2016). The experience of competence and autonomy is fundamental to intrinsic motivation and sustained interest. SDT suggests that an optimal environment for fostering intrinsic motivation—referred to as an informational environment—must satisfy individuals’ needs for autonomy and competence. These two psychological needs significantly influence self-motivation; when individuals are in autonomy-supportive environments, they are more likely to internalize values and regulations positively and engage in self-directed, intrinsically motivated actions (Shalley & Gilson, 2017). Competence, in particular, provides individuals with a sense of control over their environment and a drive to develop new skills (Van Den Broeck et al., 2016). In the workplace, competent employees actively seek out challenging tasks and, when their need for competence is met, they experience a profound sense of accomplishment (Deci et al., 2001). This, in turn, enhances their ability to tackle complex challenges and fosters their engagement in creative behaviors. Consequently, both autonomy and competence serve as key drivers of self-motivation and creative performance.

Furthermore, prior research has consistently established a positive association between competence and creative behavior (Choi et al., 2021; Rego et al., 2012). Employees who are more involved in organizational decision-making processes tend to develop a stronger sense of competence, which in turn serves as a crucial foundation for enhancing creative behavior (Choi et al., 2022). Y. Yang et al. (2016) demonstrated that increased competence leads to improvements in employees’ creative behaviors. Similarly, Thao and Kang (2018) argued that competence mediates the relationship between servant leadership and followers’ creative behavior. Additionally, Choi et al. (2022) highlighted that procedural justice fosters employees’ competence, and this improvement in ability subsequently enhances their creative behavior. Therefore, this study proposes the following hypotheses:

**Hypothesis** **3.** *Competence positively moderates the relationship between autonomy and creative behavior; that is, the positive relationship between autonomy and creative behavior is stronger when employees’ competence is high.*

**Hypothesis** **4.** *Competence moderates the indirect relationship between self-sacrificial leadership and creative behavior through autonomy, meaning that the indirect effect of self-sacrificial leadership on creative behavior through autonomy is stronger when employees have high competence.*

**Figure 1:**
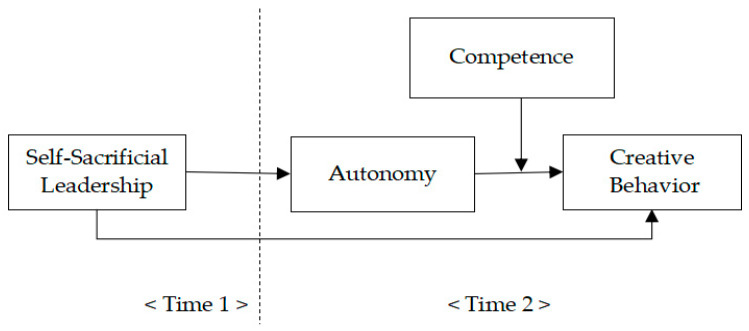
Research model.

Figure 1 illustrates the research model.

## 3. Methodology

### 3.1. Sample Collection Procedure and Characteristics

This study targeted employees in the Chinese service industry. A random sample of 472 service employees was selected from 3 service companies located in Hebei Province: a large department store, a property management company, and a driving school. Prior to the survey, respondents were informed of the research objectives, procedures, and their right to withdraw at any time, as well as the potential benefits and disadvantages of participation. Subsequently, they were asked to sign an informed consent form based on the provided information, and data were collected solely from those who provided their consent. Moreover, all data were anonymized and securely stored in a separate electronic repository under the supervision of the principal investigator.

To minimize the potential for common method bias, the survey was conducted in two waves, with a one-month interval between them. In the first wave, participants assessed self-sacrificial leadership and control variables, and in the second wave, they reported their autonomy, competence, and creative behavior. In August 2024, Wave 1 was administered to 472 participants, yielding 422 valid responses, resulting in a response rate of 89.4%. In September 2024, Wave 2 was conducted with 422 respondents from the first wave, obtaining 302 valid responses and achieving a response rate of 63.9% relative to the initial 472 participants (see Appendix A for further details).

Of the 302 respondents, 82.5% were female, and 17.5% were male. Large department stores and real estate companies constituted the primary sample for this study. As part of the service industry, these sectors exhibit employment characteristics that are particularly conducive to female workforce participation, with frontline sales positions predominantly occupied by women. Consequently, female participants accounted for 82.5% of the sample population. Although the gender distribution in the sample is imbalanced, it reflects the current gender composition of employees in China’s service industry. Their average age was 37.5 years (SD = 8.5). Regarding their highest educational qualification, 41.1% had a high school education, 31.1% had a college education, 20.2% had a middle school education, and 7.6% had a bachelor’s degree. None of the respondents had a master’s degree or higher. Because service jobs do not require much specialized knowledge, the education levels of the respondents matched the current education levels of Chinese service industry employees. Their average tenure at their current company was 5.5 years (SD = 4.8).

In this study, employees’ tenure varied, reflecting coexistence of new and experienced employees in real-world organizations. The variation in the duration of working with a leader may be related to leadership mobility within organizations, such as promotions and departmental transfers. Additionally, the wide range of interaction frequency reflects differences in the necessity of leader–subordinate interactions across various job roles. For instance, in some roles, frequent interaction with leaders is essential, whereas in others, such as independent or specialized positions, interaction frequency may be lower. These characteristics of the sample may be typical of organizations in rapidly growing and highly dynamic industries, such as China’s service sector, where constant changes and workforce heterogeneity are prevalent.

### 3.2. Measurements

The variables used in this study were gauged using a five-point Likert scale (1 = Strongly Disagree to 5 = Strongly Agree). The original measurement questions were written in English, and a back-translation process was conducted. A bilingual translator who lives in the United States and is fluent in both Chinese and English translated the original English items into Chinese. Then, a separate independent translator back-translated the items into English to ensure consistency with the original wording. In addition, three organizational behavior experts reviewed the translated scales to assess conceptual alignment and linguistic appropriateness (see Appendix B) (Brislin, 1980).

#### 3.2.1. Self-Sacrificial Leadership

Self-sacrificial leadership was measured using three items developed by De Cremer et al. (2006). Example items are “My leader engages in activities that involve considerable self-sacrifice in pursuit of organizational goals” and “My leader is a person who shows a lot of self-sacrifice”. Cronbach’s alpha was 0.90.

#### 3.2.2. Autonomy

Autonomy was measured using three items developed by Spreitzer (1995). Example items are “I have a great deal of autonomy in deciding how to do my work” and “I have considerable opportunities for independence and freedom in the way I do my work”. Cronbach’s alpha was 0.86.

#### 3.2.3. Competence

Competence was measured using three items developed by Spreitzer (1995). Example items are “I am confident in my ability to perform my job” and “I have acquired the skills needed to perform my work”. Cronbach’s alpha was 0.92.

#### 3.2.4. Creative Behavior

Creative behavior was measured using three items developed by Neubert et al. (2016). Example items are “I generate creative ideas in my work” and “I am innovative in my work”. Cronbach’s alpha was 0.96.

#### 3.2.5. Control Variables

To ascertain the relationships among the variables presented in our research model more clearly, we referred to previous studies that dealt with similar research variables. Accordingly, we employed age, sex, education level, tenure, length of time working with the leader, and frequency of interaction between employees and the leader as control variables (Bernerth & Aguinis, 2016; Goodwin et al., 2009).

## 4. Results of Data Analysis

### 4.1. Analytical Strategy

Confirmatory factor analysis (CFA) was performed to validate the research model’s legitimacy. To test our research hypotheses using hierarchical regression analysis, STATA 18.0 (Stata Corp., College Station, TX, USA) was employed. The analysis to verify the mediated and modulated mediated effects using bootstrapping followed Hayes’ recommendations (Hayes, 2013, 2018; Hayes & Rockwood, 2017).

### 4.2. Common Method Bias

The survey was conducted in two staggered phases to mitigate the likelihood of common method bias. For the analyzed sample (n = 302), Harman’s single-factor test indicated that the first factor accounted for 23.68% of the variance, which is well below the critical threshold of 50%. While we acknowledge the inherent limitations of this methodology, these findings suggest a relatively low likelihood of significant common method bias in our study (Fuller et al., 2016; Podsakoff et al., 2003).

### 4.3. Descriptive Statistics and Correlations

Table 1 presents the means, standard deviations, correlations, and Cronbach’s alpha values. Consistent with the hypotheses, significant correlations were observed among the research variables.

### 4.4. Confirmatory Factor Analysis

Table 2 presents the results of the CFA conducted to validate the construct validity of the research variables. The chi-squared to degrees of freedom ratio was 2.23, which is below the threshold value of 3.00. The comparative fit index and Tucker–Lewis index were 0.961 and 0.941, respectively, which closely approached the preferred benchmark of 0.950. The root mean square error of approximation stood at 0.064, well below the maximum allowable limit of 0.080. Based on these fit indices, the hypothesized four-factor model exhibited an excellent fit. After comparing the three alternative models, the four-factor structure was determined to be the best fit. All variables met the standard criteria with average variance extracted (AVE) values exceeding 0.5 and composite reliability values surpassing 0.7. The correlation coefficients between variables were below the square root of the AVE (Fornell & Larcker, 1981). Moreover, all the standardized factor loadings of the predictor variables were greater than 0.50.

### 4.5. Hypothesis Testing

Hierarchical multiple regression analyses were used to test Hypotheses 1 and 3; Hypotheses 2 and 4 were tested using the bootstrapping method (Hayes, 2013, 2018; Hayes & Rockwood, 2017). Upon examining Hypothesis 1, as shown in Model 3 in Table 3, a significant positive relationship was observed between self-sacrificial leadership and creative behavior.

For Hypothesis 2, an indirect effect analysis was performed using 10,000 bootstrapping iterations. As detailed in Table 4, the results indicated that the 95% confidence interval did not encompass 0, confirming Hypothesis 2.

**Figure 2:**
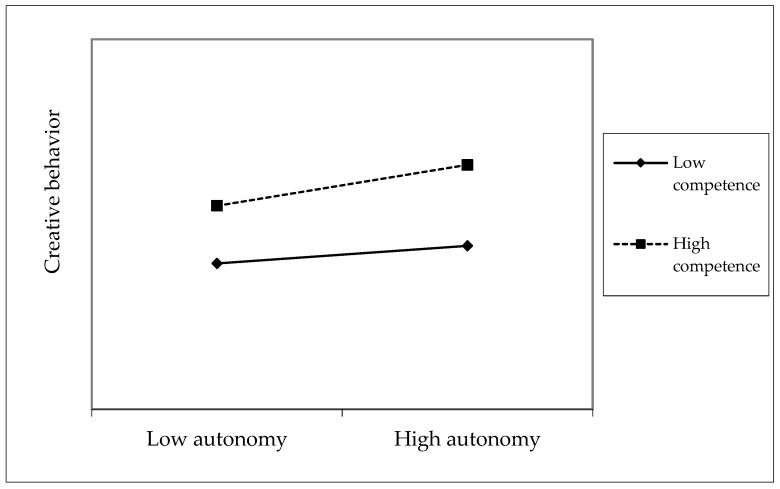
Moderating effect of competence on the relationship between autonomy and creative behavior.

Hypothesis 3 posited that competence moderates the relationship between autonomy and creative behavior. In Model 6 of Table 3, a significant interaction was identified between autonomy and competence. Model 6 presented a significantly enhanced explanatory capability compared to Model 5. To address potential multicollinearity issues and ensure a clearer analysis of the moderation effect, we applied grand mean centering to the predictor variables included in the interaction terms. Figure 2 illustrates the direction and slope of the moderation effect.

Subsequent simple slope tests revealed that the relationship between self-sacrificial leadership and autonomy was significant at a high level (+1 SD) of competence but not at a low level (−1 SD) (see Figure 2). Thus, the moderating effect was confirmed (Aiken & West, 1991).

Hypothesis 4 (moderated mediation effect) was tested using a bootstrapped indirect effect test with 10,000 iterations, following previous methods (Hayes, 2013, 2018; Hayes & Rockwood, 2017). The mediating effect (i.e., the influence of self-sacrificial leadership on creative behavior via autonomy) was examined at high (+1 SD) and low (−1 SD) competence levels. As shown in Table 5, the mediating effect was significant at the high competence level (with a confidence interval excluding 0; lower limit of confidence interval (LLCI) = 0.01, upper limit of confidence interval (ULCI) = 0.07) but not at the low level (confidence interval includes 0; LLCI = −0.00, ULCI = 0.03). Thus, Hypothesis 4 was supported.

## 5. Discussion

### 5.1. Theoretical Contributions

This study contributes to the literature on self-sacrificial leadership and creative behavior by offering several theoretical insights.

First, unlike previous studies that primarily focused on transformational and servant leadership as key drivers of creative behavior (Gumusluoglu & Ilsev, 2009; Yoshida et al., 2014), this study provides empirical evidence on how self-sacrificial leadership influences creativity. Through personal sacrifice and role modeling, self-sacrificial leadership enhances employees’ identification with and sense of responsibility toward organizational goals, thereby fostering creative behavior. By demonstrating a distinct pathway through which self-sacrificial leadership promotes creativity, this study broadens the existing leadership literature.

Second, this study identifies autonomy as a mechanism of mediating in the relationship between self-sacrificial leadership and creative behavior. Previous studies have shown that transformational leadership fosters creativity through relational identification, while empowering leadership enhances creativity through psychological empowerment (Qu et al., 2015). This study empirically verifies that self-sacrificial leadership enhances employees’ autonomy, which in turn facilitates creative behavior. Grounded in self-determination theory (SDT), this study highlights the pivotal role of autonomy in strengthening intrinsic motivation, thus presenting a novel psychological pathway through which leadership influences employee creativity.

Third, this study examines the moderating role of competence in the relationship between self-sacrificial leadership and creative behavior. While prior research has largely focused on personal traits, leader characteristics, and leader–follower relationships as moderators, this study provides empirical evidence on how competence functions as a key psychological factor that amplifies the effects of leadership on creative behavior (Hughes et al., 2018). The findings indicate that when employees have high competence, the motivational impact of self-sacrificial leadership becomes more pronounced. This insight extends the understanding of competence, demonstrating that it serves not only as a precursor to creativity but also as a crucial moderating factor that enhances the effectiveness of leadership.

Finally, this study expands the application of SDT in leadership and creativity research by demonstrating that autonomy and competence play central roles in explaining the influence of self-sacrificial leadership on creative behavior. While previous studies predominantly relied on social exchange theory to explain leadership effects on creativity, this study adopts SDT to reveal how leadership fosters creativity by stimulating employees’ intrinsic motivation. By incorporating autonomy and competence as key psychological mechanisms, this study lays a strong theoretical foundation for future research, further extending the application boundaries of SDT in leadership and creative behavior studies.

### 5.2. Practical Implications

First, this study provides directional guidelines for fostering employees’ creative behavior. The results of this study inform managers that self-sacrificial leadership can stimulate employees’ creative behavior. Organizations can strengthen leaders’ self-sacrificial leadership through training and learning to better stimulate team members’ potential to achieve the common development of individuals and organizations.

Second, the results can be used to optimize human resource management. By understanding the role of autonomy and competence in this context, organizations can take appropriate measures, such as empowering employees and providing development opportunities to enhance employees’ intrinsic motivation and self-confidence and promote creative behavior. This means that by fostering employees’ autonomy and competence, organizations can enhance their identification with and response to self-sacrificial leadership, which, in turn, will enhance their creative behaviors.

Third, the results will contribute to enhancing leaders’ cognitive level and leadership skills. By understanding the mechanism through which self-sacrificial leadership influences employees’ creative behavior, leaders can refine their leadership and management practices. By identifying the determinants of creativity, leaders can adopt more effective strategies to foster innovation within their teams.

Fourth, the results will help shape a positive organizational culture that is conducive to innovation. Self-sacrificial leadership conveys care and trust to employees, helps cultivate mutual respect and a humane organizational atmosphere, shapes a good working environment, and stimulates employees’ initiative and creative potential.

Finally, the findings of this study indicate that self-sacrificial leadership can foster employees’ creative behavior. To effectively develop and apply self-sacrificial leadership, organizations should implement the following practical strategies. Organizations should design leadership development programs that equip managers with the skills to practice self-sacrificial leadership. Leadership training courses can incorporate mentorship and role-modeling exercises, enabling leaders to demonstrate commitment and fairness in their interactions with employees.

At the policy level, fostering an environment that supports employee autonomy is essential. Establishing decision-making delegation systems can empower employees with greater authority over their tasks. Additionally, expanding flexible work policies allows employees to determine their work methods and goals with greater independence. Integrating self-sacrificial leadership into performance evaluation and reward systems can further reinforce its impact. Organizations can introduce assessment criteria that recognize and reward managers who embody self-sacrificial leadership, encouraging a workplace culture rooted in dedication and commitment. By adopting these strategies, organizations can enhance the effectiveness of self-sacrificial leadership and cultivate an environment that encourages and sustains employees’ creative behavior.

### 5.3. Research Limitations and Future Research Directions

First, this study may have been affected by common method bias. The data were collected from three service industry units, and although methods such as anonymous completion and one-to-one supervision were adopted, they may still have had some influence on the study’s data and conclusions. To address the limitations of self-reported surveys, this study employed a two-wave, time-lagged data collection approach. The results of Harman’s single-factor test indicated a low likelihood of common method bias. However, we acknowledge that common method bias cannot be entirely ruled out. Additionally, the demographic composition of the sample is somewhat skewed toward female respondents, reflecting gender distributions within certain industries and job roles. This may limit the generalizability of the findings. Future research should consider employing multi-source data collection methods and expanding the study sample to encompass a broader range of industries and occupations, thereby enhancing the robustness and applicability of the findings.

Second, this study was based on a survey of the creative behaviors of Chinese service industry workers and was affected by the sample size and sampling location, so the study may not be sufficiently representative. Further research should endeavor to expand the sample size while further differentiating the survey areas and conducting research from entry points such as the east, middle east, west, south, north, south, coast, and inland. More research value can be added to future studies by analyzing the differences between countries represented by different countries or continents.

Third, because self-sacrificial leadership is always deficient in the study of antecedent variables, self-sacrificial leadership can be studied thematically in future research to provide richer material.

Fourth, this study has a limitation in that it does not account for the influence of cultural factors. Therefore, when interpreting the findings, it is important to consider the boundary condition that this research was conducted within the Northeast Asian cultural context. Cultural dimensions such as collectivism versus individualism, power distance, masculinity versus femininity, and uncertainty avoidance are essential in understanding how the impact of self-sacrificial leadership may vary across different national and cultural settings. Future research should conduct cross-national comparative studies to examine these aspects more comprehensively. Doing so would enhance the generalizability of the findings and provide a more nuanced understanding of the effects of self-sacrificial leadership.

Finally, self-sacrificial leadership can have a positive impact on employees but can also be a “double-edged sword” with negative impacts if the antecedents are over an appropriate level. Therefore, in the future, we can study the nonlinear relationship of self-sacrifice leadership’s impact on employees.

### 5.4. Conclusions

This study aimed to uncover the mechanism of the interaction effect between autonomy and competence. According to SDT, employees influenced by self-sacrificial leadership are more likely to show stronger autonomy and intrinsic motivation and be more actively engaged in work, thereby increasing the occurrence of creative behavior. The empirical results also showed that competence, as a positive emotion, positively regulates the impact of autonomy on creative behavior, and this impact becomes stronger when competence is higher. This study not only provides empirical evidence for the impact of self-sacrificial leadership on creative behavior through autonomy but also explains the mechanism of the interaction between autonomy and competence, providing specific insights for organizational change, innovation and human resource management.

## Figures and Tables

**Table 1:** Means, standard deviations, correlations, and reliabilities.

Variables	Mean	SD	1	2	3	4	5	6	7	8	9	10	11
1. Gender	0.18	0.38	-										
2. Age	37.51	8.54	−0.50 ***	-									
3. Education	3.26	0.87	0.04	−0.26 ***	-								
4. Job level	1.29	0.45	−0.05	−0.08	0.10 *	-							
5. Tenure	5.50	4.84	−0.01	0.32 ***	−0.06	0.13 **	-						
6. Time Working with Leader	3.91	3.30	−0.07	0.34 ***	−0.07	0.08	0.65 ***	-					
7. Interaction Frequency	4.92	5.96	−0.06	0.11 *	0.11 *	0.22 ***	0.04	0.02	-				
8. SL	3.49	1.04	−0.10 *	0.02	−0.01	−0.02	0.03	0.10 *	0.06	(0.90)			
9. CO	3.38	0.70	0.27 ***	−0.30 ***	0.06	0.13 **	0.06	0.02	0.09	0.12 **	(0.86)		
10. AU	3.21	0.68	0.23 ***	−0.30 ***	0.00	0.08	0.02	−0.02	0.08	0.14 **	0.78 ***	(0.92)	
11.CB	2.97	0.74	−0.10	0.09	−0.03	0.03	0.14 **	0.10*	0.01	0.16 ***	0.59 ***	0.54 ***	(0.96)

Note. n = 302; * *p* < 0.05, ** *p* < 0.01, *** *p* < 0.001; the values in parentheses denote Cronbach’s alphas. Gender: female = 0, male = 1; age: year; education = final level of education: 1 = primary school graduate, 2 = junior high school graduate, 3 = high school graduate, 4 = college graduate, 5 = university graduate, 6 = post-graduate, 7 = Ph.D. holder. Job level: 1 = staff, 2 = manager. Tenure: organizational tenure (year), time working with leader (year). Interaction Frequency: employee–leader interaction frequency (time); SL = self-sacrificial leadership; CO = competence; AU = autonomy; CB = creative behavior.

**Table 2:** Confirmatory factor analysis results.

Model	χ2 (df)	CFI	TLI	RMSEA	Δχ2(Δdf) 4
Research model (4 factor)	237.91 (92) ***	0.96	0.94	0.07	
Alternative model 1 (3 factor) ^1^	951.55 (101) ***	0.77	0.69	0.17	713.64 (9) ***
Alternative model 2 (2 factor) ^2^	1336.13 (109) ***	0.67	0.58	0.19	1098.22 (17) ***
Alternative model 3 (1 factor) ^3^	2429.18 (138) ***	0.38	0.27	0.24	2191.27 (46) ***

Note. n = 302; *** *p* < 0.001; CFI = comparative fit index; TLI = Tucker–Lewis index; RMSEA = root mean square error of approximation; 1 3 factor: SL + CO, AU, CB; 2 2 factor: SL + CO + AU, CB; 3 1 factor: SL + CO + AU + CB; 4 Chi square difference for each model reflects its deviation from the four-factor model. SL = self-sacrificial leadership; CO = competence; AU = autonomy; CB = creative behavior. X^2^(df) = Chi-square statistic (degrees of freedom).

**Table 3:** Hierarchical multiple regression.

Variables	AU		CB			
	Model 1	Model 2	Model 3	Model 4	Model 5	Model 6
Gender	0.09	0.11	−0.08	−0.15 **	−0.19 ***	−0.17 ***
Age	−0.29 ***	−0.29 ***	−0.00	0.17 **	0.20 ***	0.17 *
Education	−0.09	−0.08	−0.02	0.03	0.01	−0.00
Job level	0.04	0.05	0.01	−0.02	−0.05	−0.03
Tenure	0.08	0.09	0.15 *	0.09	0.07	0.08
Time Working with Leader	0.02	0.00	−0.02	−0.02	−0.04	−0.04
Interaction Frequency	0.05	0.05	−0.01	−0.04	−0.04	−0.04
SL		0.15 **	0.14 *	0.05	0.04	0.05
AU				0.62 ***	0.23 ***	0.21 **
CO					0.53 ***	0.49 ***
AU*CO						0.12 *
R^2^	0.12	0.14	0.03	0.05	0.48	0.49
adj R^2^	0.10	0.12	0.01	0.02	0.46	0.48
F_inc_		7.51 **		153.22 ***	59.16 ***	7.02 **

Note. n = 302; * *p* < 0.05, ** *p* < 0.01, *** *p* < 0.001; Standardized coefficients are reported. SL = self-sacrificial leadership; CO = competence; AU = autonomy; CB = creative behavior. R^2^ = R-squared, coefficient of determination. adj R^2^ = adjusted R-squared, adjusted coefficient of determination. Finc = incremental F-statistics.

**Table 4:** Results of bootstrapped indirect effect test.

	Dependent Variable: CB			
Mediator	Indirect Effect	SE	95% CI	
			LL	UL
AU	0.06	0.02	0.02	0.11

Note. n = 302; number of bootstrapping iterations = 10,000; LL = lower limit, UL = upper limit; AU = autonomy; CB = creative behavior.

**Table 5:** Results of bootstrapped conditional indirect effect test.

		Dependent Variable: CB			
Moderator	Indirect Effect	Coefficient	SE	95% CI	
				LL	UL
CO	Low (Mean − 1 SD)	0.01	0.01	−0.00	0.03
	High (Mean + 1 SD)	0.03	0.02	0.01	0.07

Note. n = 302; number of bootstrapping iterations = 10,000. CO = competence; CB = creative behavior. LL = lower limit, UL = upper limit.

## Data Availability

The raw data supporting the conclusions of this article will be made available by the authors without undue reservation to any qualified researcher.

## Abbreviations

The following abbreviations are used in this manuscript:

OCB	Organizational citizenship behavior.
SDT	Self-determination theory.
AVE	Average variance extracted.
LLCI	Lower limit of confidence interval.
ULCI	Upper limit of confidence interval.
X^2^(df)	Chi-square statistics (degrees of freedom).
R^2^	R-squared, Coefficient of Determination.
adj R^2^	Adjusted R-squared, adjusted coefficient of determination.
Finc	Incremental F-statistics.

## Appendix B. Measurement

**Self-sacrificial leadership (α = 0.90) (De Cremer et al., 2006)**

1. In pursuing organizational objectives, engages in activities involving considerable self-sacrifice.
2. Takes high personal risk for the sake of the organization.
3. My boss is somebody who shows a lot of self-sacrifice.

**Competence (α = 0.86) (Spreitzer, 1995)**

1. I am confident about my ability to do my job.
2. I am self-assured about my capabilities to perform my work activities.
3. I have mastered the skills necessary for my job.

**Autonomy (α = 0.92) (Spreitzer, 1995)**

1. I have significant autonomy in determining how I do my job.
2. I can decide on my own how to go about doing my work.
3. I have a considerable opportunity for independence and freedom in how I do my job.

**Creative behavior (α = 0.96) (Neubert et al., 2016)**

1. I generate creative ideas at work.
2. I promote and champion ideas to others.
3. I am innovative at work.
